# Planned Abscopal Effect with Concurrent Pembrolizumab and Ablative Radiotherapy to Pulmonary Metastasis: A Case Report and Review of the Literature

**DOI:** 10.3390/curroncol31120573

**Published:** 2024-12-04

**Authors:** Yu-Ting Lee, Chien-Chin Chen, Hsiya Chao, Chih-Chia Chang, Cheng-Yen Lee

**Affiliations:** 1 Division of Hematology and Oncology, Department of Medicine, Ditmanson Medical Foundation Chiayi Christian Hospital, Chiayi 60002, Taiwan; 07623@cych.org.tw; 2 Department of Biomedical Sciences, National Chung Cheng University, Chiayi 62100, Taiwan; 3 Department of Pathology, Ditmanson Medical Foundation Chia-Yi Christian Hospital, Chiayi 60002, Taiwan; 07265@cych.org.tw; 4 Department of Biotechnology and Bioindustry Sciences, College of Bioscience and Biotechnology, National Cheng Kung University, Tainan 701401, Taiwan; 5 Department of Cosmetic Science, Chia Nan University of Pharmacy and Science, Tainan 717301, Taiwan; 6 Ph.D. Program in Translational Medicine, Rong Hsing Research Center for Translational Medicine, National Chung Hsing University, Taichung 402202, Taiwan; 7 Department of Biomedical Engineering, Chung Yuan Christian University, Taoyuan 320314, Taiwan; 06261@cych.org.tw; 8 Department of Radiation Oncology, Ditmanson Medical Foundation Chiayi Christian Hospital, Chiayi 60002, Taiwan; 07229@cych.org.tw; 9 Ph.D. Program in Advanced Manufacturing Systems, Advanced Institute of Manufacturing with High-tech Innovations, National Chung Cheng University, Chiayi 62100, Taiwan

**Keywords:** abscopal effect, radiotherapy, immune check point inhibitor, pembrolizumab

## Abstract

Most abscopal effects are reported as sporadic and unpredictable events following radiotherapy at symptomatic sites. Herein, we report a case in which a planned abscopal effect was induced following deliberate radiotherapy and concurrent systemic immunotherapy. A 53-year-old man with a combined positive score ≥10 developed extensive metastatic bladder cancer after progressing on conventional chemotherapy. Extensive metastases were identified in his liver, lungs, and bones. He later had four cycles of single-agent pembrolizumab and planned hypofractionated radiotherapy at an ablative dose to selected metastatic lung tumors and developed complete remission of disease even when pembrolizumab was discontinued. This is a clear demonstration that the abscopal effect could be harnessed in a systematic manner with a combined positive score and aggressive local radiotherapy.

## 1. Introduction

Immunotherapy is an integral part of the treatment of urothelial carcinoma of the bladder. In the case of early-stage bladder cancer, intravesical immunotherapy with bacillus Calmette–Guérin is widely used. When disease extends beyond the confines of the pelvic muscle layer or spreads to other parts of the body, systemic immunotherapy may be offered. The most common systemic immunotherapies are immune checkpoint inhibitors (ICIs), which are engineered molecules that block proteins on cancer cells or immune cells that prevent the immune system from attacking cancer cells. Pembrolizumab, in particular, is a highly selective, humanized monoclonal antibody against programmed death 1 (PD-1) that disrupts and impedes inhibitory signals in T cells, therefore augmenting the systemic anti-tumor response, pivotal to the treatment of metastatic and locally advanced bladder cancer [1].

Radiotherapy is known to regulate the tumor microenvironment and thus augment ICI’s tumoricidal effect [2]. In the most dramatic setting, systemic anti-tumor response, known as the abscopal effect, may be elicited. It is defined as radiation-induced immune-mediated tumor regression at lesions distant from the irradiated target. Its mechanism is a field of great interest [3]. Whereas past reports on abscopal effects with and without ICIs were mostly unexpected treatment outcomes, demonstrating the sporadic nature of the phenomenon, the development of modern image-guided radiotherapy (IGRT) has enabled precision delivery of tumoricidal doses while sparing normal tissues from treatment-related toxicity [4,5]. This advancement has materialized radiotherapy as an in situ vaccine. Herein, we have taken a systematic approach to harness the abscopal effect in a patient with metastatic bladder cancer whose disease progressed on cisplatin-based chemotherapy.

## 2. Case Report

**Figure 1:**
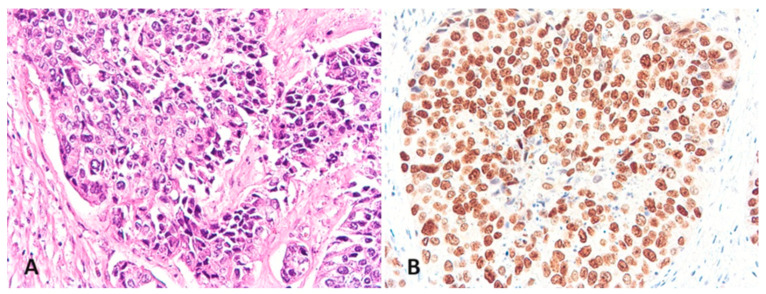
H&E stain of bladder urothelial carcinoma at 200× (**A**) and GATA-3 (**B**). The combined positive score was ≥10.

A 53-year-old Asian diabetic male former smoker had total cystectomy for bladder cancer at age 50 after three years of recurrent local disease and repeated local tumor resection with intravesical epirubicin instillation. Prior to the surgery, intractable hematuria and extravesical extension of the bladder tumor were noted. Tumor response to local epirubicin instillation was modest, with final pathology yielding ypT4aN1 in April 2021 (Figure 1). Programmed death ligand 1 (PD-L1) expression was ≥10 as assessed by combined positive score (CPS) on the pharmDx^®^ assay (Toronto, ON, Canada). The proportion of tumor area occupied by PD-L1-expressing tumor-infiltrating immune cells was 1%. Shortly after the surgery, the patient developed metastasis in multiple bone sites and had 3600 cGy in 12 fractions delivered to a painful soft tissue mass in the pelvic bone as palliation.

**Figure 2:**
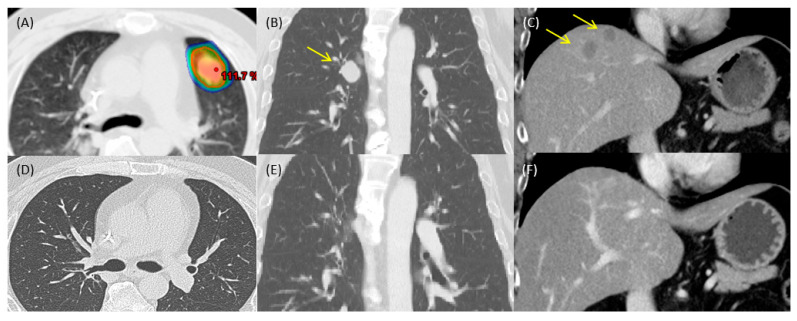
Abscopal effect following concurrent pembrolizumab and hypofractionated radiotherapy. Radiotherapy isodose curves of the irradiated lung tumor (**A**) and representative tumors in the lung (**B**) and liver (**C**). Complete response of the irradiated tumor (**D**) and subsequent remission of unirradiated tumors in lung (**E**) and liver (**F**).

He was put on a CG regimen with cisplatin (50 mg/m^2^ day 1) and gemcitabine (800 mg/m^2^ day 1 and day 8) as first-line treatment for metastatic disease. There was symptomatic relief to the regimen, and he was off chemotherapy after six cycles with remission of lesions on bone scintigraphy. Progression of the disease was noted eight months later, with multiple metastatic lesions arising in his liver, lungs, and bones. At the peak of his disease burden, the extent of skeletal metastasis included the lumbar spine, right humeral head, and iliac wing. Meanwhile, ten and six metastatic lesions were identified in his lungs and liver, respectively (Figure 2B,C). No metastasis in the brain was noted. Palliative radiotherapy with 3000 cGy in 10 fractions was delivered to the painful lumbar spine with symptomatic relief. The patient lost a considerable amount of body weight, from 83 kg to 80 kg, as the disease progressed. There were no associated respiratory symptoms. Liver function remained adequate.

Systemic treatment was shifted to National Health Insurance (NHI), which reimbursed pembrolizumab (200 mg triweekly). Meanwhile, the patient was well informed of his condition and realized the potential outcomes of radiotherapy. Participating healthcare workers adhered to the principles of medical ethics. Written informed consent was obtained from the patient before the commencement of all treatments. Hypofractionated radiotherapy to an asymptomatic metastatic tumor in the left upper lung with 6000 cGy in 10 fractions was offered concurrently with the second cycle of pembrolizumab and continued on pembrolizumab to a total of 4 cycles by May 2022 (Figure 2A). The patient was simulated on 4D CT. Planning target volume included the gross tumor and its respiratory trajectory. Treatment planning was on Eclipse version 10.0^®^ (Palo Alto, CA, USA). Radiotherapy was carried out on weekdays 1, 2, 4, and 5, with daily cone beam CT image-guided radiotherapy on a Varian Ix^®^ (Palo Alto, CA, USA) linear accelerator.

Complete remission of the metastatic tumors was noted following completion of the assigned ICIs. The patient could not be put on maintenance pembrolizumab for the NHI reimbursement policy. The patient had improved general condition, and recovery in weight was significant (from 80 kg to 88 kg). Satisfied with the improvement in performance status and quality of life, the patient even returned to his former work. Apart from minor pulmonary fibrosis at the irradiated site (Figure 2D), there was no treatment-related toxicity. Following radiography showed complete remission of disease (Figure 2D–F).

**Figure 3:**
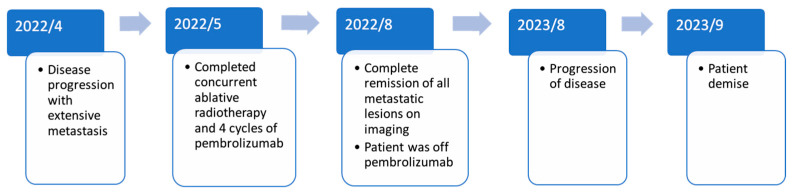
Milestones in the clinical course of the patient who developed durable abscopal effect following concurrent ablative radiotherapy and four cycles of pembrolizumab.

In August 2023, weeks before his demise, he presented to our emergency service with a fever and shortness of breath. CT confirmed disease relapse, with multiple lesions in the lung, liver, and skeleton. Palliative radiotherapy was delivered to the painful left femur at 2000 cGy/5 fx. Systemic therapy with paclitaxel was offered. Nevertheless, he succumbed to disease after three cycles of paclitaxel. Overall, he has been alive for 15 months since completing pembrolizumab concurrently with hypofractionated radiotherapy. The institutional review board has granted permission to report this case. The clinical course is summarized in Figure 3.

## 3. Discussion

The abscopal effect remains a highly desired but unpredictable systemic anti-tumor response. Most cohorts on observed abscopal effects were limited to histopathologies other than urothelial carcinoma and as results of unexpected treatment outcomes. For example, in a systematic review of case reports on the abscopal by Abuodeh et al., urothelial carcinoma was not reported [4]. Meanwhile, the number of case reports on the abscopal effect is increasing with the introduction of ICIs [6].

A literature review was performed on PubMed employing the query string: ((pembrolizumab) AND (abscopal)) AND (radiotherapy). After limiting the results to case reports, 23 articles remained. The authors went through them manually. Table 1 summarizes case reports on the abscopal effect with pembrolizumab limited to urothelial carcinoma. Meanwhile, results of ongoing trials on multimodality treatment of urothelial carcinoma, including but not limited to pembrolizumab, are highly anticipated [7].

The decision to deliver ablative radiotherapy was a planned one rather than a random process. Despite the inherent bias associated with a case report, a considerable amount of evidence-based medicine already exists. CPS may be both prognostic and predictive. The patient was offered radiotherapy because he had a CPS score ≥ 10. CPS score has been shown to be a relevant prognostic factor for those diagnosed with metastatic urothelial carcinoma. CPS ≥ 10 also correlates with superior survival and treatment response [1]. In a case report for the abscopal effect for mesothelioma with pembrolizumab, a CPS score = 30 was confirmed histopathologically [10].

There are several mechanisms that precipitate the augmentation of anti-tumor immune response. First, radiation acts as an in situ vaccine that induces immunogenic tumor cell death and the release of tumor-specific antigens [11]. Secondly, tumor cells that have survived radiation may develop immune susceptibility. Next, radiation eradicates local immunosuppressive lymphocytes and alters the tumor microenvironment, resulting in local effects on endothelial cell expression that facilitate immune cell trafficking and immune cell activation [12]. It is also worth mentioning that radiation should be tailored to systemic therapy to augment systemic anti-tumor effects [13].

In the absence of a universally accepted biochemical indicator for the abscopal effect. Most published data on abscopal effects in the presence of ICIs were based on clinical observations that may be either limiting or indirect. However, there are important distinctions between the systemic effect of ICIs and the abscopal effect. The abscopal effect is characterized by the following findings: First, the delay between radiotherapy and the onset of tumor regression is typical of the abscopal effect. In a systematic review of abscopal effects, the median interval between radiotherapy and the onset of abscopal effect was two months (range 0–24 months) [4]. For our patient, systemic tumor response was noted after completion of four cycles of assigned pembrolizumab, each cycle three weeks apart. Secondly, the persistence of disease control after cessation of pembrolizumab also consolidates the presence of an immunogenic anti-tumor effect. Our patient had remarkable survival (15 months) despite presenting with hepatic metastasis. Such survival would not be expected with pembrolizumab alone, as shown in the Bellmunt study that patients with hepatic metastasis did not benefit from pembrolizumab monotherapy [1]. In addition to that, clinical trials have demonstrated unfavorable response rates of hepatic metastasis to ICIs [14,15]. This surplus in overall survival time in our study without the continuation of pembrolizumab for our patient is highly suggestive of the synergistic effect of pembrolizumab and radiotherapy.

Orchestrating ICIs and radiotherapy is an important issue. It has been shown that radiation can be either immunostimulatory or immunosuppressive. Preclinical and clinical studies have shown an increase in anti-tumor response to high-dose hypofractionation as compared to conventional fractionation. Supporting discoveries include, but are not limited to, (1) high-dose hypofractionation-induced immunogenic tumor cell necrosis enhances cross-presentation of tumor antigen by dendritic cells to CD8^+^ T-cells in draining lymph nodes and (2) survival of cross-primed anti-tumor CD8^+^ T-cells from cell death by radiation, as hypofractionated radiotherapy is likely to be completed before the migration of these radiosensitive lymphocytes to infiltrate tumor mass [16]. In the absence of symptomatic lesions, we have chosen an asymptomatic lung tumor for maximal radiation dose escalation. Unlike metastasis in the liver, dose escalation for lung tumors is far more achievable in the absence of radiation-sensitive organs, such as the liver, without developing significant treatment-related toxicity. In a pooled analysis of two trials on pembrolizumab for metastatic non-small cell lung cancer with and without ablative radiotherapy (the majority delivered to pulmonary tumors), toxicity in the combination arm was unremarkable [5]. In addition to that, the patient already had two prior palliative radiotherapies that did not yield a tumor response. Therefore, the abscopal effect following another radiation at a palliative dose seemed doubtful.

The relatively small volume of the irradiated pulmonary tumor is also ideal for hypofractionated radiotherapy to 6000 cGy (biological equivalent dose_10_ = 100). Treatment was deliberately interrupted between every two treatments to mitigate the toxic effect of radiotherapy on migrating immune lymphocytes. Concurrent delivery of radiotherapy appears to deliver the best chance of an abscopal effect [5,13]. Our approach complements most publications in which radiotherapy was directed to symptomatic sites.

## 4. Conclusions

The abscopal effect was considered a sporadic and random event. Advancements in radiation delivery have made radiotherapy a tangible in situ cancer vaccine beyond a means of symptomatic relief. In this study, systemic anti-tumor response was observed following combination pembrolizumab and local ablative radiotherapy in a patient with extensive metastatic urothelial carcinoma of the bladder. This case report demonstrates not only that the abscopal effect with concurrent pembrolizumab can be planned but also that radiotherapy remains relevant in modulating systemic immune response.

## Figures and Tables

**Table 1:** Reports on the abscopal effect with pembrolizumab on urothelial carcinoma.

Authors	Year of Publication	Number of Cases	Primary Site	Radiotherapy Regimen	Note
This study	2024	1	Bladder	6000 cGy/10 fx	Radiotherapy to asymptomatic site
Kawanishi et al. [8]	2023	1	Bladder	5200 cGy/8 fx	Complete response to brain metastasis
Ishiyama et al. [9]	2020	1	Right renal pelvis	3000 cGy/10 fx	Radiotherapy after pembrolizumab

## Data Availability

The data presented in this study are available on request from the corresponding author due to limitations in patient consent.
